# Phytochemical Composition, Antioxidant and Antiproliferative Activities of Defatted Sea Buckthorn (*Hippophaë rhamnoides* L.) Berry Pomace Fractions Consecutively Recovered by Pressurized Ethanol and Water

**DOI:** 10.3390/antiox9040274

**Published:** 2020-03-25

**Authors:** Lijana Dienaitė, Audrius Pukalskas, Milda Pukalskienė, Carolina V. Pereira, Ana A. Matias, Petras Rimantas Venskutonis

**Affiliations:** 1 Department of Food Science and Technology, Kaunas University of Technology, Radvilėnų pl. 19, LT-50254 Kaunas, Lithuania; lijana.dienaite@gmail.com (L.D.); audrius.pukalskas@ktu.lt (A.P.); milda.skemaite@ktu.lt (M.P.); 2 IBET—Instituto de Biologia Experimental e Tecnológica, Food & Health Division Apartado 12, 2780-901 Oeiras, Portugal; carolina.pereira@ibet.pt (C.V.P.); amatias@ibet.pt (A.A.M.)

**Keywords:** defatted sea buckthorn pomace, pressurized liquid extraction, antioxidant capacity, antiproliferative activity, flavonoids

## Abstract

This study aimed at valorisation of sea buckthorn pomace (SBP) for the production of extracts containing valuable bioactive compounds. For this purpose, SBP defatted by supercritical CO_2_ was subjected to consecutive fractionation with pressurized ethanol and water, which yielded 11.9% and 4.8% of extracts, respectively. The extracts were evaluated for their antioxidant potential, phytochemical composition and antiproliferative effects against cancer cells. Water extracts exhibited remarkably higher values in Folin-Ciocalteu assay of total phenolic content, oxygen radical absorbance capacity (ORAC), ABTS^●+^/DPPH● scavenging and cellular antioxidant activity (CAA) assays and more efficiently inhibited proliferation of HT29 cells at non-cytotoxic concentrations measured in non-tumoral Caco2 cells. Among 28 detected and 21 quantified phytochemicals, flavonols with the structures of isorhamnetin (five compounds), quercetin (three compounds), kaempferol (three compounds) glycosides and catechin (six compounds) were the most abundant in the extracts. In conclusion, the applied method of fractionation of SBP produces promising natural antioxidant complexes with antiproliferative properties that could find potential applications in nutraceuticals, functional foods and cosmeceuticals.

## 1. Introduction

Processing of horticultural crops generates large amounts of by-products, which nowadays are used inefficiently or even discarded as a waste. It is estimated that globally 30–50% of agro-food materials (approximately 1.3 billion tons per year) are wasted, while fruits and vegetables constitute 44% of the total losses [1]. Consequently, the residues of fruits and vegetables processing represent a serious disposal problem for the industry; on the other hand, they are cheap sources of valuable nutrients and other materials, which may find various applications.

The scopes of R&D and commercialization in the area of bio-refining of agro-food processing by-products have been increasing over the last decades. However, large amounts of berry pomace rich in bioactive compounds, retained after juice pressing, are still insufficiently valorised for a wider industrial implementation of their processing technologies and commercialization of the products obtained. Numerous studies demonstrated that berry processing by-products may contain higher amounts of valuable nutrients than the whole fruits or their main products [2]. Therefore, a detailed knowledge on by-products composition and physicochemical properties is essential for developing preferable methods of their recovery and further application in foods and other products.

Sea buckthorn (*Hippophaë rhamnoides*) berries (SB) are not consumed as fresh fruits; however, they have become popular as raw materials of jams, beverages, candies, juices, etc. [3]. SB have been widely studied; their chemical composition and health benefits have been reported in numerous original articles and reviews [4]. The fruits are well known as a rich source of dietary antioxidants belonging mainly to the class of phenolic compounds, primarily proanthocyanidins, gallocatechins and flavonol glycosides [5,6]. To date, most SB-related studies have focused on health effects of juice and polyphenolic extracts produced from the whole fruits [7,8,9], while SB pomace (SBP) have been less investigated. The majority of studies focused on the isolation of tocopherol-rich lipophilic fraction from the seeds [10,11,12]. Issartier et al. [13] applied solvent free microwave-assisted extraction of antioxidants from SB press-cakes, while Varshneya et al. [14] recovered antioxidants from seedless pomace with methanol, water and their mixture. More recently, SBP and seeds were fractionated by multistep biorefining procedure [15]. However due to a lack of comprehensive and systematic valorisation studies, large amounts of SBP are discarded as a waste, thus, causing the loss of a significant fraction of valuable nutrients and biologically active compounds.

Consequently, recovery of polyphenols and other nutrient-rich extracts from SBP and development of the standardized functional ingredients is of great interest for food, nutraceutical and cosmeceutical industries. Biological berry matrix is highly heterogeneous and complex; therefore selection of effective extraction/fractionation processes is an important step in separation and recovery of bioactive compounds from the pomace. Moreover, nowadays in order to avoid undesirable changes of sensitive bioactive compounds and considering high environmental and toxicological requirements for food grade substances, green separation techniques are preferred to conventional liquid-solid extraction with hazardous organic solvents. From this point of view, supercritical fluid extraction with carbon dioxide (SFE-CO_2_), pressurized liquid extraction (PLE) with ethanol (EtOH) and water (H_2_O) have gained popularity in recent years [16,17]. It was demonstrated that SFE-CO_2_ is an effective method for recovery of lipophilic berry compounds, while extraction with EtOH and H_2_O produces the extracts containing higher polarity constituents such as phenolics, sugars and others [18,19,20,21]. The majority of polar plant origin polyphenolic compounds are strong radical scavengers and may inhibit oxidative processes in food and, possibly, provide some defense against damaging effects of excessive radical species, which may form in the cells due to the oxidative stress. Numerous studies have supported the hypothesis that dietary antioxidants may reduce the risk of oxidative stress related diseases and disorders and thereby might provide anticancer, anti-aging, antimicrobial, anti-inflammatory, anti-neurodegenerative and other health beneficial effects [22].

Bioactivities of dietary antioxidants could be measured by the in vitro extracellular and cellular assays. Many studies reported that the values measured by the chemical assays, e.g., widely used free radical (DPPH•, ABTS^•+^) scavenging, oxygen radical absorbance capacity (ORAC), ferric reducing antioxidant power (FRAP) and others, often do not correlate with the results obtained by the more physiologically relevant methods both in vitro and in vivo. [23,24]. A Caco-2 cell model has been reported to be a simple and useful system for investigating bioavailability of food phytochemicals by determining the uptake of the main compounds. To improve biological relevance of antioxidant activity results the cellular antioxidant activity (CAA) method was developed [25].

The aim of the present work was to expand our knowledge on the possibilities of recovery of the defatted by SFE-CO_2_ SBP polyphenolics by consecutive PLE with green solvents and valorisation of the extracts by the assessment of their phytochemical composition, antioxidant capacity and antiproliferative activities. ORAC, DPPH, ABTS and CAA assays were conducted to evaluate the extracellular and cellular antioxidant activities, while human epithelium colon cancer cells HT29 and human colon cancer cells Caco-2 were employed to test the antiproliferative activity and cytotoxicity of obtained extracts. The results are expected to serve in developing valuable natural ingredients for functional foods, nutraceuticals and cosmeceuticals.

## 2. Materials and Methods

### 2.1. Chemicals and Cells

Human Caco-2 and HT29 cell lines were purchased from DSMZ (Braunschweig, Germany) and ATCC (Manassas, VA, USA), respectively. The cell culture medium and supplements were purchased from Invitrogen (Gibco, Paisley, UK). Phosphate buffered saline was obtained from Sigma-Aldrich (St. Louis, MO, USA) and cell viability was assessed using a CellTiter 96^®^ AQueous One Solution Cell Proliferation Assay (Promega, Madison, WI, USA).

The Folin-Ciocalteu reagent, 6-hydroxy-2,5,7,8-tetramethylchroman-2-carboxylic acid (Trolox), 2,2-diphenyl-1-picrylhydrazyl radical (DPPH•, 98%), gallic acid, KH_2_PO_4_, KCl, NaCl, formic acid (98%), 2,2′-azino-*bis*-3-ethylbenzothiazoline-6-sulfonic acid (ABTS, 98%), K_2_S_2_O_8_, 2′,2′-azo-*bis*-(2-amidinopropane) dihydrochloride (AAPH), HPLC grade and LS-MS grade acetonitrile were obtained from Sigma-Aldrich (Darmstadt, Germany). Disodium fluorescein, Na_2_HPO_4_·2H_2_O and ethanol (99.9%) were from TCI Europe (Antwerp, Belgium), Riedel-de-Haen (Seelze, Germany) and Scharlau (Barcelona, Spain), respectively. Na_2_CO_3_, 2′,7′-dichlorofluorescin diacetate (DCFH-DA), quercetin (95%) were from Sigma-Aldrich (St. Quentin Fallavier, France). Ultra-pure water was produced in a Simplicity 185 system (Millipore, Billerica, MA, USA). Analytical grade methanol and acetone were purchased from StanLab (Lublin, Poland). The standards used for UPLC analysis (malic acid, izorhamnetin, quinic acid, rutin, citric acid) were from Supelco Analytical (Bellefonte, PA, USA), catechin, epigallocatechin from Extrasynthese (Genay Cedex, France).

### 2.2. Proximate Analysis of Sea Buckthorn Pomace (SBP)

The chemical composition of SBP was determined according to the procedures established by the Association of Official Analytical Chemists [26]: moisture by drying at 105 °C to constant weight; ash by mineralizing in a muffle furnace F-A1730 (Thermolyne Corp., Dubuque, IA, USA) at 500 °C for 3 h; proteins by Kjeldahl method in a nitrogen analyzer (Leco Instruments Ltd., Mississauga, ON, Canada) using a conversion factor of 6.25; crude lipids by Soxhlet extraction with hexane for 6 h. The rest of dry matter was assigned to carbohydrates. Each determination was carried out in triplicate.

### 2.3. Sea Buckthorn Pomace Preparation and Extraction

Frozen *Hippophaë rhamnoides* SBP were obtained from local farmer, immediately freeze-dried and ground in a laboratory mill Vitek (An-Der, Austria) by using 0.5 mm size sieve (further indicated in all calculated values as dry weight powder, DWP). SBP powders were extracted by SFE-CO_2_ in a 100 mL extractor (Applied Separations, Allentown, PA, USA) to remove lipophilic fraction. PLE of defatted pomace powder (10 g) was mixed with diatomaceous earth (4 g), placed in 66 mL extraction cells and consecutively extracted with ethanol (SBP-E) and water (SBP-W) in an accelerated solvent extraction apparatus ASE350 (Dionex, Sunnyvale, CA, USA) at constant 10.3 MPa pressure and temperature (70 °C for SBP-E and 120 °C for SBP-W) using 15 min static and 90 s purge time for each extraction cycle (in total 3 cycles). EtOH was evaporated in a Rotavapor R-114 (Büchi, Flawil, Switzerland), while residual water was removed by freeze-drying in a Maxi Dry Lyo (Hetto-Holton AIS, Allerod, Denmark). The extracts were weighed and stored at −18 °C in a freezer until further analysis.

### 2.4. Total Phenolic Content (TPC) and Antioxidant Capacity Evaluation Analysis

TPC, DPPH, ABTS and ORAC assays were selected for the characterisation of SBP extracts. Detailed description of these methods is provided elsewhere [27]. Briefly, for TPC assay extract solutions were mixed with Folin–Ciocalteau reagent and 7% Na_2_CO_3_ in a 96-well microplate. The absorbance was measured at 765 nm after 30 min in a FLUOstar Omega Reader (BMG Labtech, Offenburg, Germany). TPC was expressed in mg of GAE/g dry extract weight (DWE) and DWP.

For ABTS^•+^ decolourisation 6 µL of sample were added to 294 µL of ABTS^•+^ working solution, while for DPPH• scavenging 8 μL of sample were mixed with 292 µL of DPPH• methanolic solution. The absorbance was measured in 96-well microplates using a FLUOstar Omega Reader (BMG Labtech, Ortenberg, Germany) during 30 min at 734 nm and 60 min at 515 nm for ABTS^•+^ and DPPH•, respectively. Trolox was used as a standard, antioxidant capacity of the extracts was determined from the calibration curves and the results were expressed as µM TE/g DWE and DWP. Each analysis was carried out in six replicates.

For ORAC assay 25 µL of sample and 150 µL (14 μM) fluorescein solutions were placed into the wells of a black 96-well microplate. Then the mixture was preincubated in a FLUOstar Omega Reader for 15 min at 37 °C and 25 µL of AAPH (240 mM) were pipetted into each well. The fluorescence was recorded every cycle (in total, 120 cycles) using 485 excitation and 530 emission fluorescence filters. Antioxidant curves (fluorescence versus time) were first normalized and from the normalized curves the net area under the fluorescein decay curve (AUC) was measured. The results were expressed in µM TE/g DWE and DWP.

### 2.5. Analysis of Recovered Phytochemicals

#### 2.5.1. HPLC-DPPH• Scavenging Online Analysis

HPLC analysis was performed on a Waters HPLC system (Waters Corporation, Milford, MA, USA) equipped with a Waters 996 photodiode array detector, 1525 binary pump, column oven, and Rheodyne 7125 manual injector (Rheodyne, Rohnert Park, CA, USA), using a Hypersil C18 analytical column (250 × 0.46 cm, 5 µm; Supelco Analytical, Bellefonte, PA, USA). The mobile phase was 0.4% aqueous formic acid (*v*/*v*, A) and acetonitrile (B), with a gradient elution of 95% A, then changing to 90% in 5 min, after that, in 11 min A was decreased to 84%, then in 29 min to 60% A, in 5 min to 5% A and then it was hold at 5% for 3 min and in 2 min it was returned to initial conditions and column was equilibrated for 5 min. The flow rate was 0.8 mL/min, the injection volume 20 µL, and column temperature was maintained at 30 °C. UV spectra of compounds eluted from the column was recorded in the range from 220 to 450 nm and after the UV detection freshly prepared DPPH• (6 × 10^−5^ M) solution subsequently was introduced to the main eluent flow and directed to the reaction coil (15 m, 0.25 mm ID) at a flow rate of 0.6 mL/min by using Aligent 1100 series pump (Agilent Technologies, Inc. Santa Clara, CA, USA). The decrease of absorbance at 515 nm was recorded as negative peaks by a Shimadzu SPD-20A UV detector (Shimadzu Corporation, Kyoto, Japan), which appeared due to reaction of radical scavenging compounds with DPPH•. Finally, identification of compounds was performed by using Waters Acquity UPLC system (Milford, MA, USA). Chromatographic conditions were as described above, while mass spectrometry parameters were as described under UPLC-QTOF-MS analysis condition (Waters Acquity UPLC system).

#### 2.5.2. Composition and Content of Phytochemicals (UPLC-QTOF-MS)

The extracts were analysed on a Waters Acquity UPLC system (Milford, MA, USA), comprising a MaXis 4G Q-TOF mass spectrometer, a sample manager, PDA detector, binary solvent manager and controlled by HyStar 3.2 (SR2 software, Bruker Daltonics, Bremen, Germany). The MS spectra were recorded in the range from 80 to 1200 *m/z*. The samples were eluted with a gradient of solvent A (1% formic acid in ultrapure water) and B (acetonitrile) on a 1.7 µm, 100 mm × 2.1 mm i.d. Acquity BEH C18 column (Waters) over 14 min at a flow rate of 0.4 mL/min. The injection volume was 1 µL and column temperature was maintained at 40 °C. Gradient elution was performed as follows: 95% A in 0–4 min, 95–90% A in 4–6 min, 90–70% A in 6–10 min, 70–5% A in 10–12 min, 5–95% A in 12–14 min. The MaXis 4G Q-TOF mass spectrometer used electron spray ionization (ESI) source, and the samples were analysed in a negative-ion mode. Two scan events were applied, namely full-scan analysis followed by data-dependent MS/MS of the most intense ions. The data-dependent MS/MS used −30.0 V collision energies (source voltage); capillary voltage was 4 kV; end plate offset 0.5 kV; flow rate of drying (N_2_) gas 10.0 L/min; nebulizer pressure 2.5 bar. Selected phenolics were quantified by UPLC-QTOF from calibration curves prepared using different concentrations (0.1–5 µg/mL) of isorhamnetin, rutin, quinic acid, citric acid, epigallocatechin and catechin. Concentration/peak area curves followed the following equations: catechin, y = 17.72x + 190.48; R^2^ = 0.999; rutin, y = 27.81x − 299.39; R^2^ = 0.997; isorhamnetin, y = 49.48x − 10596.35; R^2^ = 0.997; malic acid, y = 6.55x + 50.41; R^2^ = 0.999; quinic acid, y = 12.36x + 5696.40; R^2^ = 0.995; epigallocatechin, y = 15.08x − 810.644; R^2^ = 0.999; citric acid, y = 1275.96x + 282.07; R^2^ = 0.999.

### 2.6. Cell Culture and Sample Preparation

Water and ethanol SBP extracts were solubilized in DMSO (200 mg/mL) and ethanol (100 mg/mL), respectively and stored at –20 °C protected from light. Cell-based assays were performed using a maximum concentrations of solvents, 1% and 5% for DMSO and ethanol, respectively.

Cell lines were cultured in RPMI-1640 medium supplemented with 10% of heat-inactivated foetal bovine serum (FBS) and 1% penicillin-streptomycin (PS), in the case of Caco-2. Cells were maintained at 37 °C with 5% CO_2_ in a humidified incubator and routinely grown as a monolayer in 75 mL culture flasks.

### 2.7. Cytotoxicity Assay in Caco-2 Cell Monolayer

Cytotoxicity was assessed using confluent and non-differentiated Caco-2 cells as a model of the human intestinal epithelium [28]. Briefly, Caco-2 cells were seeded into 96-well plates at a density of 2 × 10^4^ cells/well and grown for 7 days with medium renewal every 48 h. At day 7, the cells were incubated with the samples diluted in RPMI culture medium supplemented with 0.5% FBS. The cells incubated only with culture medium were considered as a control. After 24 h, the cells were washed twice with PBS and their viability was assessed using CellTiter 96^®^ Aqueous One Solution Cell Proliferation Assay containing MTS reagent, according to the manufacturer’s instructions. Absorbance was measured at 490 nm using a Spark^®^ 10 M Multimode Microplate Reader (Tecan Trading AG, Männedorf, Switzerland) and cell viability was expressed of percentage of living cells relative to the control.

### 2.8. Cellular Antioxidant Activity (CAA) Assay

The CAA assay was carried out by the procedure of Wolfe and Liu [29]. Caco-2 monolayers (2 × 10^4^ cell/well) were obtained after 6 days of culture in a 96-well plate and washed twice with pre-warmed PBS (10 mM, pH 7.4, 37 °C). Then, 50 µL of PBS, sample and standard (quercetin, 2.5–20 µM) solution and 50 µL of DCFH-DA solution (50 µM) were added and incubated for 1 h at 37 °C, 5% CO_2_. Afterwards, 100 μL of AAPH (12 mM) solution were added to each well containing PBS/quercetin standards/samples, while 100 µL of PBS were added to the blank wells. Fluorescence kinetics was recorded every 5 min during 60 min by using a Microplate Fluorimeter FL× 800 (Bio-Tek Instruments, Winooski, VT, USA) using 485 nm excitation and 540 nm emission wavelengths. CAA values were expressed as µM of quercetin equivalents per g of extract.

### 2.9. Antiproliferation Assay

Antiproliferative effect of SBP extracts and standard compounds was evaluated in HT29 cells as described elsewhere [30]. The cells were seeded at a density of 1 × 10^4^ cells/well in 96-well plates. After 24 h they were incubated with different concentrations of the samples diluted in culture medium or in pure culture medium (control). Cell proliferation was measured after 24 h using MTS reagent, as mentioned above. Results were expressed in terms of percentage of living cells relative to the control.

### 2.10. Statistical Data Handling

All results are presented as means ± standard deviations (SD) and all experiments were repeated at least three times. The differences between means were evaluated by one-way ANOVA using the statistical package GraphPad Prism 6 software (GraphPad, San Diego, CA, USA) to identify significant differences by using statistical unpaired *t* test with *p* < 0.05.

## 3. Results and Discussion

### 3.1. Proximate Analysis, Total Yield and Antioxidant Capacity of SBP Extracts

Berries are generally composed of their skin, soft and fleshy pericarp, intracellular juice and seeds. The distribution of the different fractions, however, largely depends on berry cultivar and preparation method: for example, in SB, the respective mass fractions for skin and seeds were reported 31.9% and 10.7%, respectively [31]. In this study SBP was composed of seeds, skin and residual pulp. The content of crude protein in SBP was 16.74 ± 0.38% DWP, which is slightly higher than previously reported by Nuernberg et al. [32] (14.6%) and Pavlović et al. [33] (14.78%) and lower than determined Ben-Mahmoud et al. [34] (20.9%). Total ash content was 1.88 ± 0.02%, which is slightly lower than previously reported, 2.02 to 3.59% [33,34,35]. The major part of SB fruit lipids are present in their seeds, which remain in the pomace after pressing the juice. It was reported that triacylglycerols of SB pulp are composed mainly of monounsaturated and saturated fatty acids, whereas seed oil is rich in polyunsaturated fatty acids [36]. The content of lipids in the SBP investigated in our study was 20.78 ± 0.14%, while other authors in various SB fractions reported from 1.8 to 29.1% of lipids [4,37]. The moisture content in dried SBP in our study was 6.40 ± 0.18%. Other macrocomponents should consist mainly of carbohydrates.

It was reported in many studies that berry pomace retain remarkable fraction of antioxidants [2,21]; therefore, evaluation of antioxidant properties of SBP extracts was an important task of our study. Moreover, exhaustive recovery of antioxidants and other bioactives is very important for the development of effective processes for utilization of by-products. For this purpose two antioxidant potential characteristics were determined for each assay, namely antioxidant capacity of extracts (expressed for DWE) and recovery of antioxidants from the dry SBP (expressed for DWP).

For the recovery of higher polarity antioxidants, SBP were defatted by SFE-CO_2_ at 35 MPa, 60 °C. Afterwards, the residues were consecutively extracted by PLE with ‘green’ solvents ethanol and water. The total extract yield obtained by ethanol was approximately 2.5 times higher comparing to water (Table 1); however, antioxidant capacity values of SBP-W depending on assay method were 1.2–2 times higher than those of SBP-E. On the other hand, ethanol due to remarkably higher yield recovered 1.4–2.3 times higher amounts of polyphenolic antioxidants from dry plant material (DWP). It is evident, that the fractions of the highest polarity and less soluble in ethanol compounds were not recovered during the 1st PLE step and remained in the residue [19,20]. In addition, the increased temperature (PLE with water) also could foster the recovery of polyphenols due to the breakdown of the cell walls and increase of membrane permeability. Furthermore, heating also might soften the plant tissue and weaken the phenol–protein and phenol–polysaccharide interactions in the material, thus more phenolics would diffuse into the solvent [17].

### 3.2. Composition and Content of Phytochemicals

**Figure 1:**
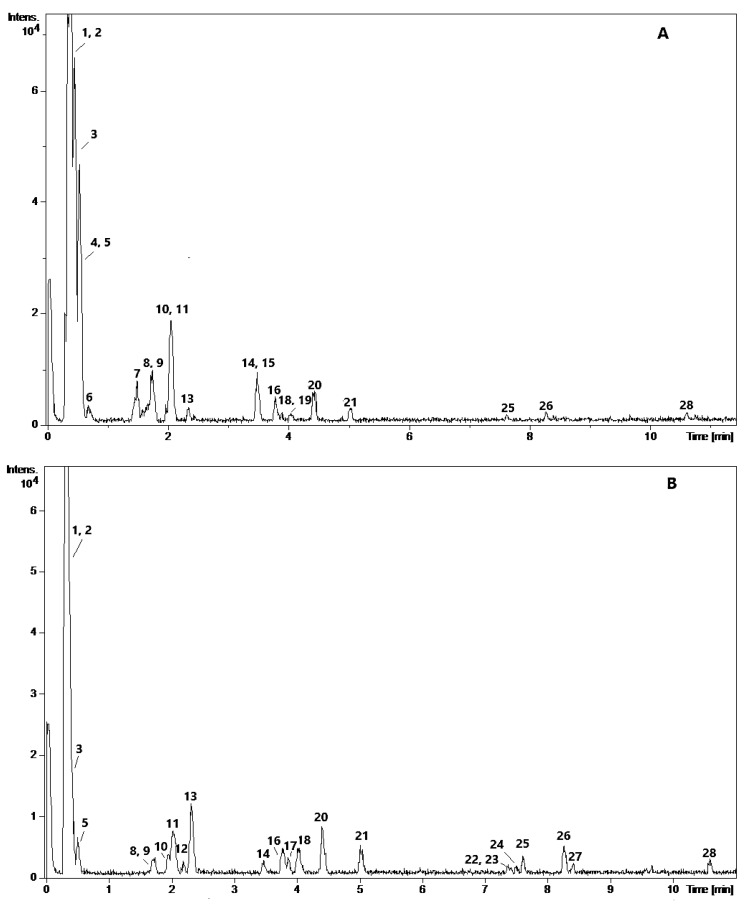
Representative UPLC-QTOF-MS chromatograms of water (**A**) and ethanol (**B**) extracts of SBP.

Phytochemical profile of SBP extracts was analyzed by UPLC-Q/TOF, while the presence of scavengers was screened by the on-line HPLC-UV-DPPH• method. The compounds were identified by calculating molecular formulas from the obtained accurate *m/z* values, assessing fragmentation patterns and comparing retention times with analytical standards and previously reported data. Twenty-eight compounds were detected by UPLC in ESI/MS mode (Table 2). The characteristic chromatograms of extracts are represented in Figure 1 (A—H_2_O, B—EtOH).

Peaks 1 and 4 gave *m/z* values of 191.0564 and 191.0199, fitting the molecular formulas C_7_H_12_O_6_ and C_6_H_8_O_7_; based on these data and by comparing with the standards the compounds were identified as quinic (1) and citric (4) acids, respectively. Compound 2 gave a molecular ion *m/z* 387 and two fragments, *m/z* 341 and *m/z* 179, indicating the loss of [M − H − CO − H_2_O]^−^ and [M − H − CO − H_2_O − 162]^−^ units from the parent molecule and suggesting 7-(α-d-glucopyranosyloxy)-2,3,4,5,6-pentahydroxyheptanoic acid. Compound 3, with *m/z* 133 fitting C_4_H_5_O_6_ and perfectly matching retention time (t_R_ 0.4) of reference compound was identified as malic acid.

Compound 5 gave an ion *m/z* 263 matching C_17_H_11_O_3_ and several fragments (Table 2) suggesting the structure of tanshinlactone or neo-tanshinlactone. These compounds are regio-isomers, differing in the positions of the lactone carbonyl and oxygen groups and cannot be differentiated by MS. In tanshinlactone, the lactone carbonyl is present at position 11 and oxygen at position 12 of the diterpenoid, while in neo-tanshinlactone, the positions are reversed. Thus, compound 5 was tentatively identified as tanshinlactone derivative [38,39].

**Figure 2:**
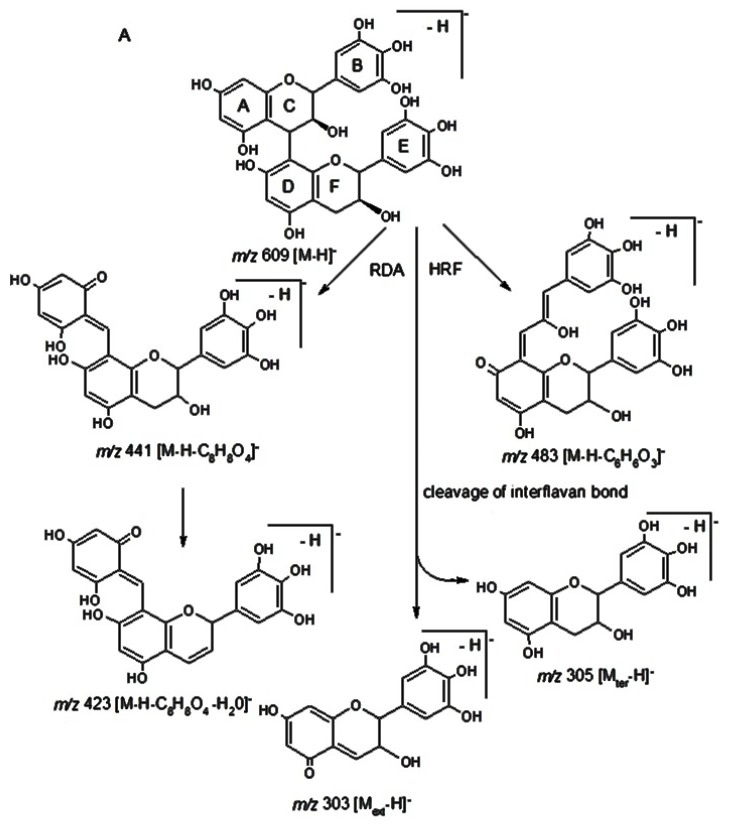

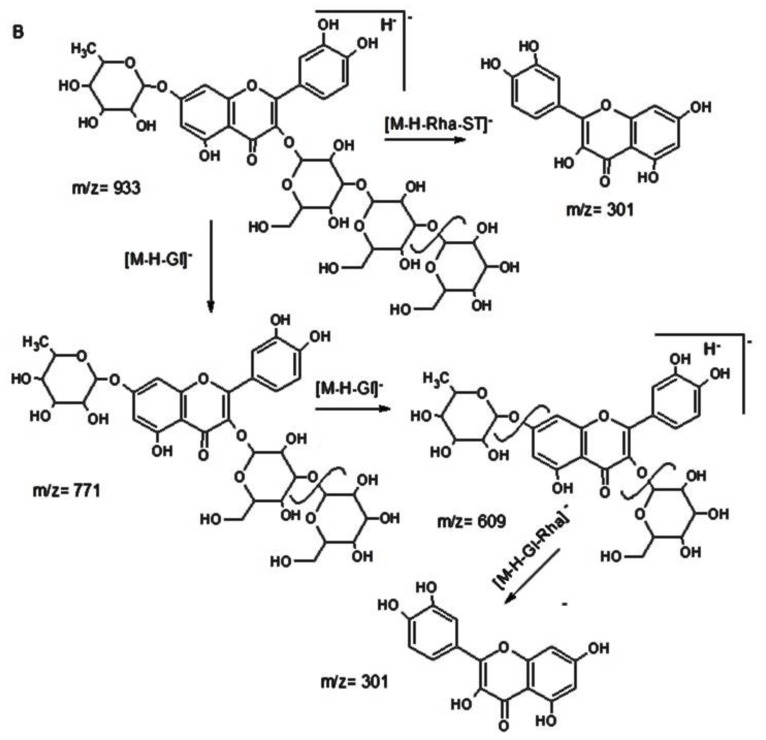
Hypothetic structures of selected daughter ions resulting from MS-MS fragmentation of the compound 6 epigallocatechin-epigallocatechin (**A**) and 15 quercetin-3-sophorotrioside-7-rhamnoside (**B**).

Compound 6 with the quasi-molecular ion *m/z* 609 (C_30_H_26_O_14_) shows the diagnostic product ion *m/z* 441, which was originated after the loss of [M − H − C_8_H_8_O_4_]^−^ unit due to cleavage of ring B from the flavan-3-ol through RDA (Retro Diels-Alder) reaction of ring C (Figure 2A). The loss of water from the diagnostic product ion *m/z* 441 produced another minor ion, *m/z* 423. Another two ions, *m/z* 591 and *m/z* 483 were the result of the loss of water from the parent ion (*m/z* 609), and [M − H − 108]^−^ unit, due to heterocyclic ring fission (HRF), respectively. Hence, the base unit of this dimer was tentatively assigned to epigallocatechin derivative. This sequence was confirmed through cleavage of the interflavanoid bond into *m/z* 305 (lower terminal subunit) and *m/z* 303 (upper subunit). The chirality at the flavan-3-ols C-3 cannot be established by MS, therefore it is impossible to distinguish whether it is gallocatechin or epigallocatechin. The connection sequence of this compound was tentatively proposed as epigallocatechin–epigallocatechin [40,41,42]. MS^2^ of the compound **7** (*m/z* 593; C_30_H_26_O_13_) in negative ESI mode yielded five main fragments, namely: *m/z* 467, 425, 407, 303 and 289. The first three ions were produced by the loss of a C_6_H_6_O_3_^−^, [M − H − C_8_H_8_O_4_]^−^ (RDA) and [M − H − C_8_H_8_O_4_ − H_2_O]^−^ (water elimination), respectively.

Finally, the direct cleavage of the interflavanoid bond gave *m/z* 289 for the base unit and *m/z* 303 for the upper terminal unit. In accordance with literature data [40,42] this compound was tentatively identified as epicatechin-epigallocatechin. The compound 8 (t_R_ 1.7 min) exhibited molecular ion *m/z* 577 (C_30_H_25_O_12_) and was assigned to epicatechin-epicatechin. Its main fragment *m/z* 425 is obtained by the loss of 152 Da due to the cleavage of flavan-3-ol ring B through RDA fission of ring C. Other characteristic fragment ion, *m/z* 407 (from RDA fission) produced by the loss of [M − H − H_2_O]^–^ from the main fragment *m/z* 425. Moreover, the loss of [M − H − C_6_H_6_O_3_]^−^ produces *m/z* 451 due to the cleavage of the two OH groups from B-ring (HRF). Further formation of *m/z* 289 and *m/z* 287 due to the cleavage of interflavan bond (from the top and the base unit) suggests that the compound **8** is a singly linked proanthocyanidin dimer [41,43]. The compound 9 gave a molecular ion [M − H]^−^, *m/z* 305 (C_15_H_13_O_7_), and the fragments of 287, 179 and 137 fitting C_15_H_11_O_6_, C_9_H_7_O_4_ and C_7_H_5_O_3_, respectively. The loss of 18, 126 and 168 amu was attributed to the loss of [M − H − H_2_O]^−^, [M − H − C_6_H_6_O_3_]^−^ and [M − H − C_8_H_8_O_4_]^−^, respectively. Based on these data and by comparing it with the standard, the compound 9 was identified as epigallocatechin [41].

MS data for the compounds 10, 12, 13 and 17 was not sufficient for their identification. Two compounds, 11 and 14 displayed a molecular ions [M − H]^−^ with *m/z* 289 (C_15_H_14_O_6_) and the major fragment ions, *m/z* 271, 247 and 245 due to the loss of [M − H − H_2_O]^−^, [M − H − CH_2_=CH–OH]^–^ and [M − H − HC≡C–OH]^–^, respectively. Furthermore, the presence of *m/z* 165, which may be formed due to HRF by elimination it from ring A, was confirmed by the fragment *m/z* 125; while the presence of *m/z* 137, which was formed due to RDA of C-ring fission, was confirmed by the *m/z* 151. Finally, the loss of dihydroxybenzene moiety [M − H − 109]^–^ gave *m/z* 179, which was confirmed by the presence of *m/z* 109 [M − H − 179]^–^. Hence, the compounds 11 (additionally confirmed by a standard) and 14 were identified as catechin and epicatechin, respectively [41,43]. The compound 15 displayed a molecular ion [M − H]^−^, *m/z* 933 (C_39_H_49_O_26_) and several fragment ions in MS/MS mode. The ion *m/z* 771 (C_33_H_39_O_21_) was a basic fragment arising from the loss of [M − H − glucosyl]^−^ (162 amu), which by a further loss of another one glucosyl moiety produces the fragment *m/z* 609 (C_27_H_29_O_16_). The ion *m/z* 301 can be derived by the loss of 632 amu from the basic *m/z* 933 ion or from ion 609 ([M − H − glucosyl − rhamnosyl]^−^). Thus, 15 was tentatively identified as quercetin-sophorotrioside- rhamnoside (Figure 2B) [44]. The fragmentation pattern of 16, 20 and 21 with *m/z* 771, 755 and 785, respectively, was similar: the loss of 146, 326 and 470 amu may be attributed to the loss of [M − H − rhamnosyl]^−^, [M − H − rhamnosyl − glucosyl − H_2_O]^−^ and [M − H − rhamnosyl − 2 glucosyl]^−^ units, respectively. Furthermore, the formation of *m/z* 301, 285 and 315 indicates the presence of quercetin, kaempherol and isorhamnetin, respectively. Based on these data and previously reported results the compounds 16, 20 and 21 were assigned to quercetin-3-sophoroside-7-rhamnoside, kaempherol-3-sophroside-7-rhamnoside and isorhamnetin-3-sophoroside-7-rhamnoside, respectively [5,45,46].

The compound 18 with *m/z* 427, (C_17_H_32_O_12_) and fragments *m/z* 249, 191, formed by the subsequent loss of hexose and [M − H − 2 × CO]^−^, respectively, was tentatively assigned to penta-*O*-hexoside. Molecular ion [M − H]^−^ of 19, *m/z* 917 (C_39_H_49_O_25_), and fragments *m/z* 775 [M − H − hexose]^−^, 593 [M − H − 2 × hexose]^−^ and 285 [M − H − 3 × hexose − pentose]^−^ (*m/z* of kaempferol), due to the loss of sugar and water molecules from the core skeleton suggest the structure of kaempferol-sophorotrioside-rhamnoside [44]. The compound 22 ([M − H]^–^, *m/z* 593) gave the most abundant fragment *m/z* 447 [M − H − 146]^−^, corresponding to the loss of rhamnose from the C-7 of kaempferol. The daughter ion *m/z* 431 [M − H − 162]^−^ resulting from the loss of the C-3-bonded glucose was less abundant. The higher sensitivity of the glycosidic linkage at C-7 position toward collision-induced fragmentation was also noted by Llorach et al. [47]. The ion *m/z* 285 indicates kaempferol and may be formed by the loss of rhamnosyl from *m/z* 431. Consequently, 22 was assigned to kaempferol-3-glucoside-7-rhamnoside [46]. The compound 23 with a molecular ion *m/z* 609 (C_27_H_29_O_16_) gave 463, (the loss of rhamnose) and 301 corresponding quercetin (after the loss of rutinose); *m/z* 179, 151 are typical fragments of rutin. Rutin identity was confirmed by the use of references; this flavonoid was previously reported in sea buckthorn [5,46]. In general, glycosylated flavonoids are among the major polyphenols in the berries while hexose conjugates are the most common representatives. Thus, MS/MS of 24 with [M − H]^–^, *m/z* 463.0890 (t_R_ 7.5 min) was identified as quercetin-3-hexoside: the loss of a hexosyl unit (162 amu), the fragment *m/z* 301 corresponding to deprotonated quercetin, as well as *m/z* 179 and *m/z* 151 confirm the structure of quercetin [5]. The [M − H]^−^ (*m/z* 623, C_28_H_31_O_16_) ions of 25 and 26 with *m/z* 477 and 461 indicate the loss of rhamnose, [M − H − C_6_H_10_O_4_]^−^ and glucose, [M − H − C_6_H_10_O_5_]^−^ from C-7, while *m/z* 315, [M − H − 146 − 162]^–^ is characteristic to isorhamnetin; these data enabled to tentatively identify them as isorhamnetin-glucoside-rhamnoside derivatives [46]. The glycoside 27 (*m/z* 477) exhibited the loss of 162 amu in their MS^2^ fragmentation, showing the linkage between glucosyl moiety and phenolic hydroxyl group. In case of 27, the deprotonated aglycone (isorhamnetin-H) ion at *m/z* 315 (base peak) was observed. Moreover, the product ion *m/z* 285 [M − H − CO − 2H]^–^ is attributable to the loss of 30 amu. Based on the above data the compound 27 was assigned to isorhamnetin-3-glucoside [46]. Based on the previously published data [5,45] and matching MS data with a standard, the compound 28 ([M − H]^–^, *m/z* 315) was identified as isorhamnetin.

**Figure 3:**
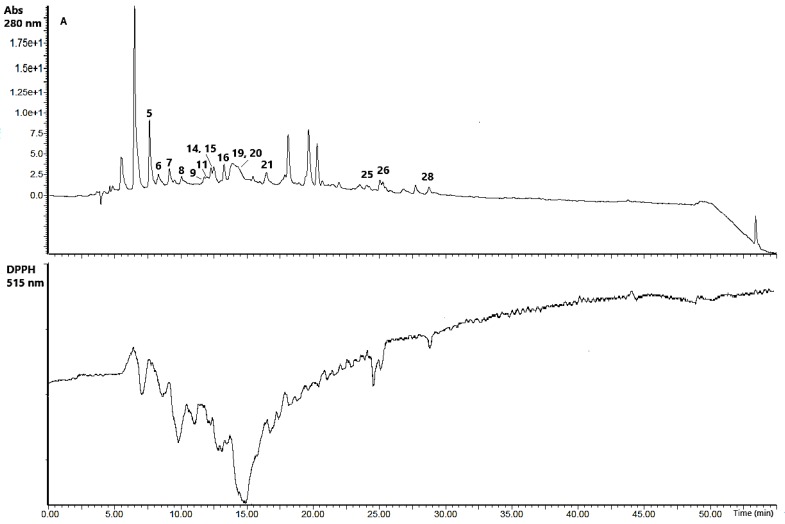

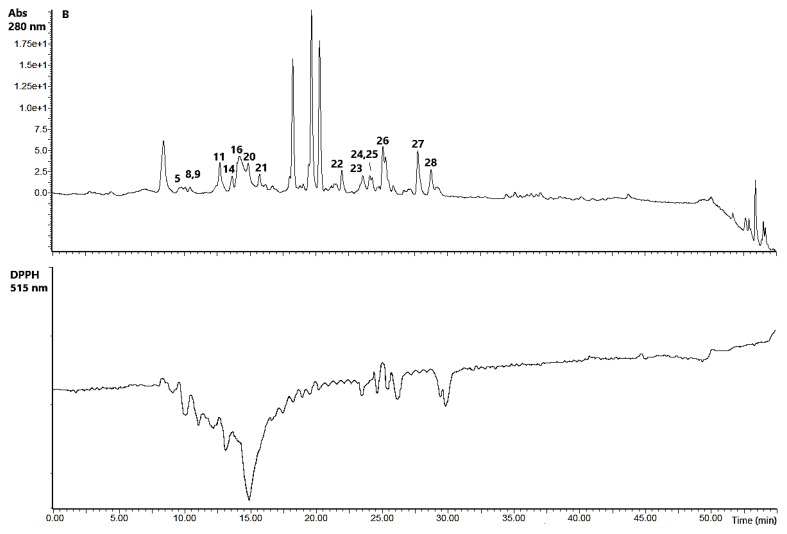
HPLC-UV-DPPH•-scavenging chromatograms of water (**A**) and ethanol (**B**) extracts of SBP.

The HPLC/UV/DPPH• chromatograms of ethanol and water extracts of SBP (negative peaks) revealed the presence of numerous radical scavenging compounds (Figure 3). It may be assumed that proanthocyanidins (6–9), flavan-3-ols (11, 14), flavonol glycosides (15, 16, 19–22, 24–27), flavonols (23, 28) terpenoid (5) were the most active radical scavengers in the investigated extracts.

In order to evaluate the potential of SBP as a source of valuable phytochemicals, the compounds were quantified by UPLC/ESI-QTOF-MS. It may be observed that SBP extracts are composed predominantly of organic acids, flavones, flavonoid monoglycosides, flavonoid diglycosides and oligomeric flavonoids (Table 3). A diversity of detected compounds were in agreement with the previously reported results [41,42,45,46,48,49,50,51]. QA and MA were the most abundant acids in the SBP (Table 4). Similar organic acid composition of SB, despite variation in acid content, which may be due to genotype, origin, harvesting time and juice processing parameters, was reported previously [50,52,53]. QA was the major compound in the all analysed samples, followed by MA; while the amounts of CA was found at lower levels. MA and QA are important contributors to the sour and astringent taste of SB [54].

Flavonoid glycosides constituted other large group of quantified in SBP compounds; 15 and 19 were quantified in SBP-W, 22 and 27 in SBP-E (Table 4). 

The content of flavonol glycosides in SBP-E was approximately 2–9 fold higher than in SBP-W. Yang et al. [48] and Ma et al. [50] reported remarkably lower amounts of Q-3-S-7-Rha and I-3-S-7-Rha in fresh SB berries than in our study, while K-3-S-7-Rha content was two times lower than reported by Guo et al. [51]. Similar results of I-3-Gl concentration reported Yang et al. [48] and Grey et al. [49] in SB berries, while concentration determined in SB berries in Ma et al. [50] and Guo et al. [51] reports were several times lower. The concentration of flavonoid diglycoside derivatives (25, 26) were in the previously reported levels in fresh SB berries [48,50] or 2-fold lower [51].

Three proanthocyanidins, 6, 7 and 8 were identified in SBP, two of them were detected only in SBP-W. The same compounds were reported previously [41,42]; however, without their quantification. Four flavones were quantified in SBP extracts and ranged in the following decreasing order: catechin > isorhamnetin > rutin > epicatechin. Guo et al. [51] determined catechin and epicatechin contents in four SB subspecies; their amounts varied from 0.82 to 4.51 and from 7.60 to 8.99 mg/100 g DW, respectively. Contents of catechin were in the range as reported, while epicatechin values were 2–6 fold lower than reported by Guo et al. [51]. Isorhamnetin content was similar to the previously reported by Rӧsch et al. [55] and Ma et al. [50], however lower than reported by Guo et al. [51]. The concentration of rutin was in agreement with Grey et al. [49]. SBP-W contained 2-fold higher EGC, than SBP-E. This compound was reported in SBP, however not quantified [41,42]. Tanshinolactone was identified and quantified for the first time in SBP; its amount was approximately 4–fold higher in SBP-W than SBP-E.

### 3.3. Antiproliferative and Cytotoxic Effects of SBP Extracts

Antiproliferative activity was assessed in HT29 cells at their exponential grow phase, while cytotoxicity was evaluated using Caco-2 cells, which share some characteristics with crypt enterocytes and therefore have been widely used to assess the effect of chemicals, food compounds and nano/microparticles [56]. 

**Figure 4:**
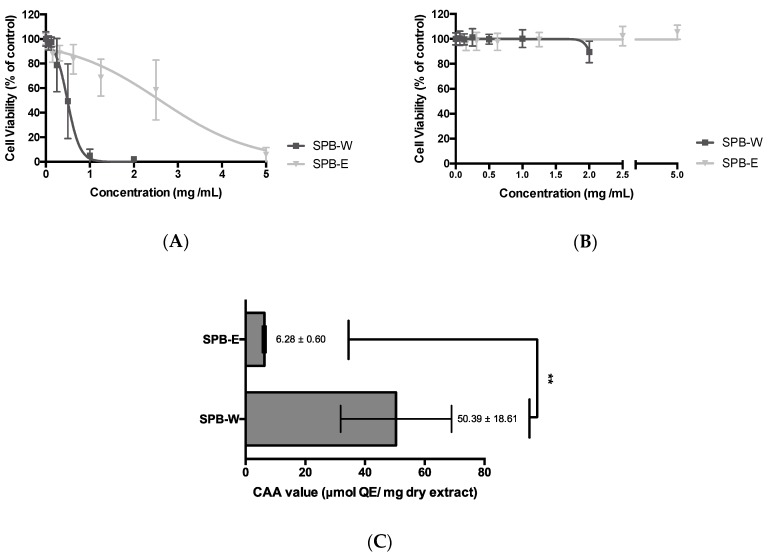
Dose-response curves of SBP extracts. Antiproliferative (**A**) and cytotoxicity (**B**) effects using HT29 and Caco-2 cell lines, respectively. Results are expressed in terms of mean ± SD performed in triplicate. Antioxidant activity of SBP extracts evaluated by CAA assay (**C**). Results are expressed as μmol quercetin equivalents (QE) per mg of dry extract of mean ± SD performed in triplicate. Unpaired *t* test was assessed with *p*-value < 0.01 (**).

Both extracts, diluted in the solvents at their maximum acceptable concentration levels, strongly inhibited cancer cell grow (Figure 4A) without cytotoxic effects on normal epithelia Caco-2 cells (Figure 4B). The bioactivities of SB extracts and plant components have been widely studied [57]. SB extracts demonstrated antiproliferative activity against colon, breast, leukemia, liver, lung and cancer cell lines [49,58,59,60,61,62]. The antitumor activity of SB can be attributed to antioxidant compounds, particularly phenolic constituents such as flavonoids catechin, kaempferol, quercetin, and isorhamnetin, which may protect cells from oxidative damage [63].

To best of our knowledge the antiproliferative activity and cytotoxicity of SBP extracts in this study are reported for the first time. Both SBP extracts inhibited HT29 proliferation in a dose-dependent manner, SBP-W being approximately 5-fold stronger (EC_50_ = 0.44 ± 0.03 mg/mL) than SBP-E (EC_50_ = 2.13 ± 0.23 mg/mL). This activity can be attributed to the presence of anticancerogenic phytochemicals (Table 2). Quercetin, kaempferol and isorhamnetin [51], catechin [62], epigallocatechin [64], procyanidins [59], tanshinlactone derivative (5) strongly suppressed cancer cell growth. In general, the antiproliferative effect of these compounds was attributed to their ability to target diverse molecular switches in carcinogen metabolism steps, including inflammation, cell proliferation, cell cycle, apoptosis and angiogenesis [65]. It is interesting noting that although SBP-E contained more flavonoid-diglycosides (I-3-S-7-Rha, Q-3-S-7-Rha K-3-S-7-Rha etc.) than SBP-W, the latter possessed stronger antiproliferative effect (Figure 4). It is in agreement with Guo et al. [51] who studied the phytochemical composition and bioactivities of the berries of four SB subspecies and found that flavonoid-diglycosides have weaker antiproliferative activity, than flavonoid-monoglycosides and aglycones. Moreover, individual pure compounds demonstrated rather weak effects, suggesting that the total antiproliferative activity of extracts may be due to the synergistic effect of phytochemicals. Most likely, better antiproliferative properties of SBP-W may be attributed to higher concentration of galloylated flavonols (6, 7, 9), and tanshinlactone derivative (5) (Table 3), which is in agreement with the previously performed studies [39,57,66,67,68].

In addition, proantocyanidins activity depend on galloylation and degree of polymerization although somewhat contradictory results were observed for this dependence. Cheah et al. [69] reported that smaller oligomers were better inhibitors, while Wang et al. [70] determined no statistically significant relationship between molecular weight and antiproliferative activity of cranberry proanthocyanidins on ovarian cancer cells SKOV-3 and OVCAR-8. It was also suggested that natural polyphenolic extracts with higher degrees of polymerization and galloylation may be more effective as antiproliferative agents than those containing monomers or oligomers [65,66]. Consequently, assuming that the monomer and oligomeric proanthocyanidins may be degraded by bacteria, the proanthocyanidins with higher molecular weight may be expected to be retained throughout the colon and exert their antiproliferative activity [71]. Moreover, in Delgado et al. [72] study catechins did not show any effects on the assayed cell lines MCF-7, Caco-2 and BxPC-3, suggesting that their absorption into those cells was limited. Tagashira et al. [73] reported that health benefits of catechin appear to be limited due to extremely low intestinal absorption. It was suggested that the presence of functional group such as gallate or pyrogallol or modification of catechins to more hydrophobic compounds could improve their bioavailability and anticancer activity. It was reported that gallates can inhibit cell growth, trigger cell cycle arrest in tumor cell lines and induce apoptosis [74]. Thus, flavonols determined in our study and containing galloyl group could be responsible for stronger antiproliferative effect of SBP-W.

The presence of diterpenoid tanshinlactone derivative, which was reported as antitumor agent [38,39,75] may also strengthen water extract activity against HT-29 cancer cells. Grey et al. [49] tested the impact of several solvents on the composition responsible for anticancer properties of SB and determined that triterpenoid ursolic acid might be more important than the polyphenols in inhibiting the cancer cell proliferation. In our case diterpenoid tanshinlactone derivative also may contribute to the activity of SBP-W.

### 3.4. Cellular Antioxidant Activity of SBP Extracts

The CAA assay assessing only those antioxidants which can penetrate living cell membrane and inhibit oxidation processes inside the cell is a more biologically relevant method than the popular chemical in vitro antioxidant capacity assays [25]. Human liver cancer HepG2 cells were previously used to determine CAA activity of crude SB extracts and its changes during digestion in gastric, intestinal and colon conditions using PBS wash (6.68 ± 0.36 μmol QE/mol phenolics) and no PBS wash protocol (17.38 ± 0.65 μmol QE/mol phenolics) [76] and also for comparing CAA of different SB subspecies [51]. SBP extracts have not been tested for their CAA previously. In our study, using Caco-2 cells SBP-W was 10-fold stronger antioxidant in CAA assay than SBP-E (Figure 4C). Consequently, SBP-W demonstrated better antioxidant potential in the all applied assays and may be considered as a better anticancer agent. It may be explained by the higher concentration of some polar hydrophilic phenolic antioxidants, which are responsible for antioxidant properties of various fruits, vegetables and berries [77,78]. For instance, water extract contained more galloylated flavonols and tanshinlactone.

## 4. Conclusions

This study proves that SB pomace is a good source of valuable phytochemicals with antioxidant capacity and cancer cell proliferation inhibitory activity. Consecutive pressurized liquid extraction of defatted SB pomace with ethanol and water enabled to produce two antioxidant-rich fractions, water extract being stronger antioxidant and more effective inhibitor of cancer cells. It may be assumed that among the 21 phytochemicals quantified in the extracts galloylated flavonols and tanshinlactone derivatives may play an important role in antioxidant and inhibitory activities of water fraction.

## Figures and Tables

**Table 1:** Yield, phenolic content and antioxidant capacity of SBP extracts.

Assay	Material	SBP-E	SBP-W
ORAC, μM TE/g	DWE	294.1 ± 6.53 ^a^	371.8 ± 8.31 ^b^
	DWP	35.26 ± 2.41 ^a^	15.84 ± 0.75 ^b^
ABTS^•^^+^, μM TE/g	DWE	268.5 ± 7.10 ^a^	323.9 ± 10.33 ^b^
	DWP	32.19 ± 1.22 ^a^	13.80 ± 2.36 ^b^
DPPH•, μM TE/g	DWE	102.3 ± 4.31 ^a^	205.0 ± 6.62 ^b^
	DWP	12.27 ± 0.51 ^a^	8.73 ± 0.33 ^b^
TPC, mg GAE/g	DWE	65.61 ± 4.80 ^a^	98.10 ± 2.01 ^b^
	DWP	7.87 ± 0.31 ^a^	4.71 ± 0.43 ^b^
Yields, %		11.91 ± 0.03 ^a^	4.80 ± 0.19 ^b^

Values represented as mean ± standard deviation (*n* = 5); a,b: the mean values followed by different superscript letters indicate significant differences between SBP-E and SBP-W for the same assay (*p* < 0.05). The extracts isolated with ethanol and water are further referred by the abbreviation composed of the short name of sea buckthorn pomace (SBP) and first letter of used solvent (E-ethanol; W-water).

**Table 2:** Identification of phenolic compounds in SB pomace extracts by UPLC-Q/TOF.

Peak No.	Compound	Abbrevation	Molecular Formula	t_R_ (min)	m/z, [M − H]^−^	SBP-E	SBP-W	MS Fragments
1	Quinic acid ^a,d^	QA	C_7_H_12_O_6_	0.3	191.0564	+	+	85; 93; 127; 173
2	7-(α-d-Glucopyranosyloxy)-2,3,4,5,6-pentahydroxyheptanoic acid ^b,c,d^	-	C_13_H_24_O_13_	0.3	387.1145	+	+	179; 341
3	Malic acid ^a,d^	MA	C_4_H_6_O_6_	0.4	133.0144	+	+	89; 115; 133
4	Citric acid ^a,d^	CA	C_6_H_8_O_7_	0.5	191.0199	−	+	43; 71; 115
5	Tanshinlactone derivative	TL	C_17_H_12_O_3_	0.5	263.0710	+	+	127; 153; 171; 217; 245
6	(e)Gallocatechin-(e)Gallocatechin ^b,d^	(e)GC-(e)GC	C_30_H_26_O_14_	0.7	609.1254	−	+	303; 305; 423; 441; 483; 591
7	(e)Catechin-(e)Gallocatechin ^b,c,d^	(e)C-(e)GC	C_30_H_26_O_13_	1.5	593.1295	−	+	289; 303; 407; 425; 467 285; 307; 429; 447
8	(e)Catechin-(e)Catechin ^b,d^	(e)C-(e)C	C_30_H_26_O_12_	1.7	577.1351	+	+	287; 289; 407; 425; 451
9	Epigallocatechin ^a,b,d^	EGC	C_15_H_14_O_7_	1.7	305.0665	+	+	137; 179; 287
10	Unknown	-	C_21_H_32_O_10_	2.0	443.1919	+	+	153; 201
11	Catechin^a,b,d^	C	C_15_H_14_O_6_	2.1	289.0716	+	+	109; 125; 137;151; 165; 179; 245; 247; 271
12	Unknown	-	C_12_H_22_O_9_	2.2	309.1190	+	−	97; 119; 161; 191; 263
13	Unknown	-	C_12_H_22_O_9_	2.3	309.1193	+	+	97; 119; 161; 191; 263
14	Epicatechin ^b,d^	EC	C_15_H_14_O_6_	3.5	289.0719	+	+	109; 125; 137;151; 165; 179; 245; 247; 271
15	Quercetin-3-sophorotrioside-7-rhamnoside ^b,c,d^	Q-ST-Rha	C_39_H_50_O_26_	3.5	933.2503	−	+	301; 609; 771
16	Quercetin-3-sophoroside-7-rhamnoside ^b,c,d^	Q-3-S-7-Rha	C_38_H_40_O_21_	3.8	771.1991	+	+	301; 445; 625
17	Unknown	-	C_25_H_40_O_14_	3.9	563.2342	+	−	191; 277; 517
18	Penta-hexoside ^c,d^		C_17_H_32_O_12_	4.0	427.1818	+	+	191; 249
19	Kaempferol-3-sophorotrioside-7-rhamnoside ^b,c,d^	K-ST-Rha	C_39_H_50_O_25_	4.1	917.2557	−	+	285; 593; 755
20	Kaempferol-3-sophoroside-7-rhamnoside ^b,c,d^	K-3-S-7-Rha	C_33_H_40_O_20_	4.4	755.2044	+	+	285; 429; 609
21	Isorhamnetin-3-sophoroside-7-rhamnoside ^b,c,d^	I-3-S-7-Rha	C_34_H_42_O_21_	5.0	785.2147	+	+	315; 459; 639
22	Kaempferol-3-glucoside-7-rhamnoside^b,c,d^	K-3-Gl-7-Rha	C_27_H_30_O_15_	7.4	593.1512	+	−	285 431; 477
23	Rutin ^a,b,d^	R	C_27_H_30_O_16_	7.4	609.1453	+	−	151; 179; 301; 463
24	Q-3-hexoside ^b,c,d^	-	C_21_H_20_O_12_	7.5	463.0890	+	−	151; 179; 301
25	Isorhamnetin-glucoside-rhamnoside derivative ^b,c,d^	I-Gl-Rha	C_28_H_32_O_16_	7.6	623.1619	+	+	315; 461; 477
26	Isorhamnetin-glucoside-rhamnoside derivative ^b,c,d^	I-Gl-Rha	C_28_H_32_O_16_	8.3	623.1623	+	+	315; 461; 477
27	Isorhamnetin-3-glucoside ^b,c,d^	I-3-Gl	C_22_H_22_O_12_	8.4	477.1040	+	−	285; 315
28	Isorhamnetin ^a,b,d^	IS	C_16_H_12_O_7_	10.7	315.0508	+	+	107; 151; 243; 300

^a^ Confirmed by a standard; ^b^ Confirmed by a reference; ^c^ Confirmed by parent ion mass using free chemical database (Chemspider, PubChem); ^d^ Confirmed by MS/MS.

**Table 3:**
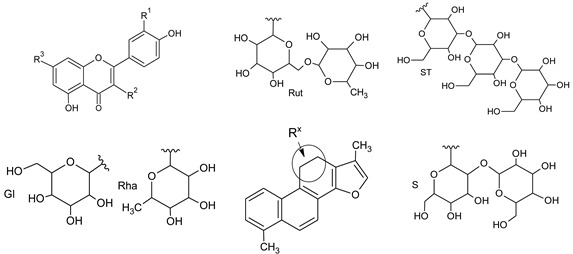
Structures of flavonol glycosides and (neo)-tanshinlactone derivative.

No.	Compound	R_1_	R_2_	R_3_
5	Tanshinlactone	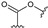	-	-
5	Neo-tanshinlactone	-	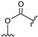	-
15	Q-3-ST-7-Rha	OH	ST	Rha
16	Q-3-S-7-Rha	OH	S	Rha
19	K-3-ST-7-Rha	H	ST	Rha
20	K-3-S-7-Rha	H	S	Rha
21	I-3-S-7-Rha	OCH_3_	S	Rha
22	K-3-Gl-7-Rha	H	Gl	Rha
23	Rutin	OH	Rut	H
25	I-3-Gl-7-Rha	OCH_3_	Gl	Rha
25	I-3-Gl-7-Rha	OCH_3_	Gl	Rha
*27*	I-3-Gl	OCH_3_	Gl	H
*28*	Isorhamnetin	OCH_3_	H	H

**Table 4:** Quantification of phytochemicals, in μg/g DWE and μg/g DWP.

Peak No.	Compounds	SBP-W		SBP-E	
		DWE	DWP	DWE	DWP
1	QA	22020 ± 698.6 ^a^	1076 ± 9.82 ^*^	48839 ± 4331 ^b^	6111 ± 104.2 ^‡^
3	MA	28842 ± 35.30 ^a^	1402 ± 1.69 ^*^	22091 ± 70.83 ^b^	2648 ± 8.44 ^‡^
4	CA	138.4 ± 4.42	6.64 ± 0.21	-	-
5	TL derivative ^g^	1906 ± 60.18 ^a^	91.52 ± 2.89 ^*^	515.9 ± 6.69 ^b^	61.45 ± 0.80 ^‡^
6	(e)GC-(e)GC ^r^	43.94 ± 3.1	2.11 ± 0.49	-	-
7	(e)C-(e)GC ^r^	118.0 ± 7.76	5.50 ± 0.40	-	-
8	(e)C-(e)C ^r^	9.53 ± 0.62 ^a^	0.27 ± 0.03 ^*^	237.76 ± 4.92 ^b^	28.32 ± 0.59 ^‡^
9	EGC	400.5 ± 5.10 ^a^	19.23 ± 0.24 ^*^	238.8 ± 1.82 ^b^	28.44 ± 0.22 ^‡^
11	C	422.4 ± 10.16 ^a^	20.28 ± 1.18 ^*^	369.6 ± 17.67 ^b^	44.02 ± 2.10 ^‡^
14	EC ^c^	150.8 ± 1.16 ^a^	6.76 ± 0.50 ^*^	123.2 ± 0.67 ^b^	14.67 ± 1.11 ^‡^
15	Q-3-ST-7-Rha ^r^	149.9 ± 6.72	17.85 ± 0.80	-	-
16	Q-3-S-7-Rha ^r^	646.7 ± 9.42 ^a^	31.04 ± 0.45 ^*^	1220 ± 39.53 ^b^	145.32 ± 4.71 ^‡^
19	K-3-ST-7-Rha ^r^	64.39 ± 7.26	3.09 ± 0.35	-	-
20	K-3-S-7-Rha ^r^	777.6 ± 14.13 ^a^	37.32 ± 0.68 ^*^	1739 ± 42.21 ^b^	207.2 ± 5.03 ^‡^
21	I-3-S-7-Rha ^r^	520.9 ± 21.45 ^a^	25.00 ± 1.03 ^*^	1166 ± 26.80 ^b^	138.9 ± 3.19 ^‡^
22	K-3-Gl-7-Rha ^r^	-	-	203.5 ± 5.42	24.24 ± 0.65
23	R	-	-	162.9 ± 7.44	19.41 ± 0.89
25	I-Gl-Rha derivative ^r^	55.23 ± 3.75 ^a^	2.50 ± 0.29 ^*^	530.2 ± 10.76 ^b^	63.14 ± 1.28 ^‡^
26	I-Gl-Rha derivative ^r^	145.2 ± 8.46 ^a^	6.66 ± 0.61 ^*^	539.4 ± 9.00 ^b^	64.24 ± 1.07 ^‡^
27	I-3-Gl ^r^	-	-	139.8 ± 0.78	16.65 ± 0.09
28	IS	71.36 ± 0.64 ^a^	3.43 ± 0.03 ^*^	195.1 ± 4.12 ^b^	23.23 ± 0.49 ^‡^

^r, c^ and ^g^ based on calibration curve obtained by using rutin, catechin and epigalocatechin, respectively; Values expressed as mean standard deviation (*n* = 3); (a, b) and (*, ‡): means not sharing common letters and symbols for the same compound are significantly different (*p* < 0.05).
